# Vitamin D and Pancreatic Cancer—An Update

**DOI:** 10.3390/cancers3010213

**Published:** 2011-01-06

**Authors:** Kun-Chun Chiang, Chun-Nan Yeh, Tai C. Chen

**Affiliations:** 1 Department of Surgery, Chang Gung Memorial Hospital and Chang Gung University, Taiwan; E-Mails: robertviolet6292@yahoo.com.tw (K.C.C.); ycn@adm.cgmh.org.tw (C.N.Y.); 2 Boston University School of Medicine, 715 Albany Street, Boston, MA 02118, USA

**Keywords:** vitamin D, pancreatic cancer, prevention, treatment, vitamin D receptor, chemoprevention, adenocarcinoma

## Abstract

The non-classical actions of vitamin D, namely anti-proliferation, pro-differentiation, immune function modulation, and anti-inflammation, have received great attention during the past decade, in particular, the potential of vitamin D analogs alone or in combination with other anticancer agents for the treatment of a variety of cancers. The association between vitamin D status and the higher incidence of many forms of cancer has suggested that vitamin D may play a role in the etiology of these types of cancer. Although it is still controversial whether this association exists for pancreatic cancer, biochemical evidence clearly indicates pancreatic cancer cells are responsive to the inhibitory effect of vitamin D and its analogs. In this review, we discuss briefly the origin and current therapy of pancreatic cancer, the history, source, metabolism and functions of vitamin D, the recent progress in the epidemiological studies of sunlight, and vitamin D status, and biochemical studies of vitamin D analogs in the prevention and treatment of pancreatic cancer.

## 1. Introduction

Pancreatic adenocarcinoma (PCA) is one of the most lethal human malignancies. Among the most common causes of cancer-related mortality, PCA ranks fourth in the Western countries and fifth worldwide [1]. In the U.S., 37,680 new cases of PCA were identified and 34,290 died from this disease in 2008 [2]. The almost 1:1 ratio of incidence to mortality clearly indicates a poor prognosis and the lethal nature of PCA, which is attributable to difficulty of early diagnosis, early local spread, distant metastasis and resistance to traditional chemotherapy and radiotherapy. The overall five-year survival rate is estimated to be in the range of 1–4%, which is much lower than the other types of cancers, such as colon, breast and prostate cancers [1]. Up to the present time, surgical extirpation has been the choice of treatment. However, the overall five-year survival rate after resection is only about 10–29% [3-5]. Moreover, at the time of presentation, 40% patients already had distant metastasis and another 40% were diagnosed with locally advanced cancer [1,2,6], which excluded them from being good candidates for resection.

Although the definite causes of pancreatic cancer still remain poorly understood, several environmental factors have been implicated. Among them, the use of tobacco has been linked to an increase in the incidence of pancreatic cancer from abundant epidemiological studies conducted since 1966 [7]. It has been reported that smokers have a greater chance of developing pancreatic cancer than nonsmokers and the risk increases with increasing frequency and length of tobacco exposure [8]. After cessation of cigarette smoking for 10 years or longer, the risk of pancreatic cancer dwindles [9]. Albeit, alcohol has been implicated as a risk factor for several types of cancers, such as the cancers of liver and esophagus, the association between alcohol and pancreatic cancer is less convincing [10,11]. A pooled analysis of 14 cohort studies reported a positive relationship between pancreatic cancer and alcohol consumption in women consuming more than 30 g of alcohol per day [12]. High caloric intake and obesity are also found to be risk factors for pancreatic cancer [13-16]. Nevertheless, fruits and vegetables failed to offer any protective benefit for pancreatic cancer in a large-scaled cohort study [17]. Others, like intake of coffee, use of aspirin, previous cholecystectomy, and history of diabetes or chronic pancreatitis, may be contributing factors for pancreatic cancer as well, although less conclusive [18-20].

While investigating the incidence of pancreatic cancer in different locations, an interesting geographical variation has been observed, that is in northern latitudes, the incidence of pancreatic cancer is three- to four-times higher than that in areas closer to equator [21]. This finding has been attributed to sunlight or ultraviolet (UV) B exposure, which is directly related to vitamin D synthesis in humans. Epidemiologic studies have shown that vitamin D status, influenced by living at high or low latitude, solar UVB exposure and dietary intake of vitamin D, is inversely associated with the incidence of some cancers such as prostate, colon and breast [22-24].

Recently, due to the dismal outcome of available chemotherapy and radiotherapy for pancreatic cancer, some new regimens or strategies have been developed for the treatment of pancreatic cancer. Here, we describe the current advances in the understanding of pancreatic cancer etiology, recent controversy on the relation between sunlight, vitamin D and pancreatic cancer incidence, the potential use of vitamin D analogs for the prevention and treatment of pancreatic cancer, and a brief history, metabolism and functions of vitamin D.

## 2. The Origin of Pancreatic Cancer and Current Therapy

Pancreatic cancer originates from the pancreatic ductal epithelium. The disease is believed to evolve from premalignant lesions to invasive cancer, combined with successive accumulation of various gene mutations during the progressive process [25]. The premalignant lesions include pancreatic intraepithelial neoplasia (PanINs), intrapancreatic mucinous neoplasia and mucinous cystic neoplasia, with PanINs being the most common and best characterized histological precursor of pancreatic cancer [26,27]. PanINs lie in small (less than 5 mm) pancreatic duct, and consist of columnar to cuboidal cells containing mucins [28,29]. Fourtier classifications are used to describe PanINs, which are PanINs-1A, PanINs-1B (low grade PanINs and refer to as flat and papillary type, respectively), PanINs-2 (intermediate grade PanINs ), and PanINs-3 (high grade PanINs ), to reflect its sequentially evolutionary process to pancreatic cancer. During the progression from low to high grade PanINs, accumulation of gene mutations is observed, which includes up-regulation of the oncogene KRAS2 [30] and down-regulation of tumor-suppressor genes, such as CDKN2A [31], TP53 [32], and DPG 4 [33]. Besides originating from premalignant lesions, pancreatic cancer may derived from a subgroup of about 1–5% of cells with stem cell properties [34,35]. Through unlimited self-renewal and asymmetric division, these pancreatic cancer stem cells lead to more un-differentiated cells. In addition, due to chemotherapy- and radiotherapy-resistant properties of pancreatic cancer stem cells, treatment of pancreatic cancer has become very difficult. Currently, the standard treatment for resectable pancreatic cancer remains surgery. However, only 20% of PCA patients are surgically operable [1,2,6]. After operation, adjuvant chemotherapy with either gemcitabine or a combination of fluorouracil and leucovorin is able to improve progression-free period and overall survival [36-38]. Combination of adjuvant chemotherapy and radiation therapy seems to increase overall survival; however, the results are not significant [39]. For unresectable pancreatic cancer, the principle of treatment is mainly palliative. The standard chemotherapy for this group of patients is gemcitamine alone [40].

## 3. Role of Vitamin D in Cancer Treatment

### 3.1. History of Vitamin D

In 1822, Sniadecki noted that children living on farms had a lower prevalence of rickets compared to the children who lived in the city of Warsaw, Poland. In 1889, Theodore Palm, a medical missionary and epidemiologist, observed that children living near equatorial areas did not have rickets and suggested sunbathing as a possible cure and strategy for rickets prevention. They both attributed their finding of geographic differences in rickets incidence to varied exposures to sunlight [41,42]. In 1918, Edward Mellanby kept dogs indoors and fed them with oats exclusively, which successfully made the animals rachitic, and this disease could be cured by cod liver oil. During that period, cod liver oil was known to treat night blindness and fracture. Mellanby did not know whether the cure of rickets was due to the newly discovered vitamin A present in cod liver oil [43] or a new substance. It was not until 1922 that McCollum clearly demonstrated that the anti-rachitic principle present in cod liver oil was a new substance and named it “vitamin D” [44]. Around the same period, Huldshinsky in 1919 discovered that children with rickets could be cured by exposure to sunlight [45]. So, there seemed to be some close relationship between sunlight exposure and vitamin D. Steenbock and Black [46] then noted that UV-irradiated food could cure rickets, which led to a great discovery later that UV light was capable of transforming one substance stored in food and skin to another form. In other words, UV light could produce vitamin D, which possesses anti-rachitic activity.

### 3.2. Source and Metabolism of Vitamin D

The sources of vitamin D for humans includes exposure to sunlight, food, and vitamin D supplement. Among them, exposure to sunlight is responsible for about 90% of vitamin D requirement [47]. Vitamin D has two forms: vitamin D_2_ and vitamin D_3_. Vitamin D_2_ is mainly synthesized from ergosterol of yeast and vitamin D_3_ is produced from 7-dehydrocholesterol of lanolin. When human skin is exposed to UV irradiation (wavelength 290–315 nm), 7-dehydrocholesterol, stored in the basal and suprabasal layers of skin [48,49], is photolyzed to form previtamin D_3_, which is then thermoisomerized to vitamin D_3_ [47,50,51]. Either vitamin D_3_ or ingested vitamin D_2_ enters the blood circulation and is carried by vitamin D binding protein (DBP) to other organs, such as the liver. In the liver, vitamin D (representing vitamin D_2_ and vitamin D_3_) is further converted to 25-hydroxyvitamin D [25(OH)D] catalyzed by vitamin D-25-hydroxylase (25-OHase) [52]. Serum 25(OH)D, the index of vitamin D status in humans, has even higher affinity for DBP than vitamin D, and therefore is also bound to DBP in the circulation. 25(OH)D is then further hydroxylated by 25(OH)D-1α-hydroxylase (1α-OHase or CYP27B1) in the kidneys to form 1α,25-dihydroxyvitamin D [1α,25(OH)_2_D], which is the most biologically active form of vitamin D. Both 25(OH)D and 1α,25(OH)_2_D can be hydroxylated by 25(OH)D-24-hydroxylase (24-OHase or CYP24A1) to form their corresponding 24-hydroxylated metabolites. Hydroxylation at carbon 24 of the vitamin D molecule by 24-OHase is the first step of the inactivation process for vitamin D [52]. However, it is now established that 1α-OHase and 24-OHase are expressed in many tissues and cells [52-54], including the expression of 1α-OHase in the pancreas [55,56]. Thus, the pancreas has the ability to active and inactivate vitamin D in an autocrine/paracrine fashion [55].

### 3.3. Functions of Vitamin D

**Figure 1.**
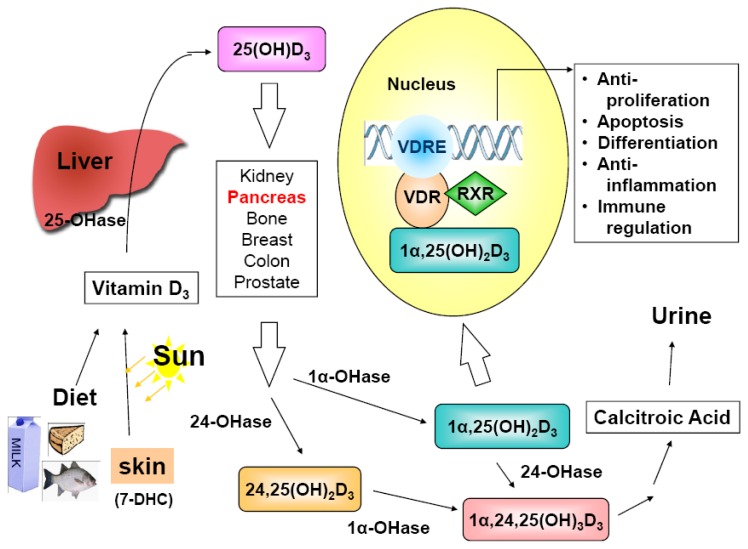
Vitamin D sources, metabolism, mechanism of action and biological activities. Vitamin D_3_ (cholecalciferol) is either derived from the diet, including supplements, or synthesized in the skin via sunlight exposure (290-315 nm) from the precursor 7-dehydrocholesterol (7-DHC). Vitamin D_3_ is initially hydroxylated in the liver by vitamin D-25-hydroxylase (25-OHase) to generate the circulating prohormone 25-hydroxyvitamin D_3_ [25(OH)D_3_]. The subsequent conversion of 25(OH)D_3_ to the active form, 1α,25-dihydroxyvitamin D_3_ [1α,25(OH)_2_D_3_], occurs in the kidneys catalyzed by a tightly regulated enzyme 25(OH)D-1α-hydroxylase (1α-OHase or CYP27B1). However, the activation may take place in many extra-renal tissues, including pancreas, bone, breast, colon, prostate, *etc.* The extra-renal synthesis of 1α,25(OH)_2_D may be one reason why serum 25(OH)D level, instead of the circulating level of the active form, 1α,25(OH)_2_D, is the index of vitamin D nutritional status. The resulting 1α,25(OH)_2_D_3_ elicits its transcriptional effects by binding to the vitamin D receptor (VDR)/retinoid X receptor (RXR) complex on vitamin D response element (VDRE) in the promoter region of vitamin D responsive genes. The cellular effects include anti-proliferation, pro-differentiation, pro-apoptosis, anti-inflammation, immune response regulation, *etc.* In addition to 25-OHase and 1α-OHase, 24-OHase (CYP24A1) also plays an important role in the vitamin D metabolic cascade, and thereby, in the regulation of vitamin D actions. The primary role of 24-OHase is to hydroxylate 1α,25(OH)_2_D_3_ and 25(OH)D_3_ to their corresponding 24-hydroxylated metabolites, the first step of vitamin D catabolic pathway to inactivate VDR ligands.

1α,25(OH)_2_D exerts its hormone-like functions through binding to vitamin D receptor (VDR), an endocrine member of the nuclear receptor superfamily [57] to regulate its target genes (Figure 1). A study, in which a Chip-sequencing method was applied to define genome-wide mapping of VDR binding, reported that VDR was bound to 2,776 genomic sites in 229 vitamin D-regulated genes [58]. Since VDR was first identified in 1979 in many tissues not known for regulating calcium and bone metabolism [59], it is not surprising that 1α,25(OH)_2_D may possess functions beyond its originally identified action on calcium homeostasis and bone mineralization. It is now well-established that 1α,25(OH)_2_D exhibits anti-proliferative, pro-differentiating, anti-inflammatory, and pro-apoptotic activities in a tissue- and cell-specific manner [55,60-62] and, so far, it has been shown to have growth inhibitory effect on prostate, colon, breast, lung, liver and pancreatic cancer cells, which express VDR [47,63-67].

### 3.4. Vitamin D and Pancreatic Cancer

As mentioned in Section 3.3, 1α,25(OH)_2_D_3_ possesses anti-tumor activity through anti-proliferative, pro-apoptotic, and pro-differentiation actions in a cell- and tissue-specific manner [60-62,64]. Regarding pancreatic cancer, 1α,25(OH)_2_D_3_ has been shown to up-regulate the expression of p21 and p27 and down-regulate the expression of cyclins A, D1, and E, leading to cell cycle arrest at G_0_/G_1_ phase [68]. However, 1α,25(OH)_2_D_3_ is known to cause hypercalcemia and hypercalciuria side effects. To overcome these lethal side effects caused by systemic administration of 1α,25(OH)_2_D_3_, several thousands of less calcemic or noncalcemic analogs of 1α,25(OH)_2_D_3_ have been synthesized and studied *in vitro* and *in vivo* animal models. Some of them have been found to have more potent anti-tumor activity mediated by cell-cycle arrest, stimulating differentiation, and/or promoting apoptosis on pancreatic cancer cells *in vitro* and in the xenograft animal model [68-73]. One of these analogs, EB-1089, has been investigated in a phase II clinical trial to treat advanced pancreatic cancer. However, the trial showed that the analog failed to prolong the survival of patients significantly [74]. In a recent published phase II clinical trial enrolling 25 advanced pancreatic cancer patients, a combination of oral 1α,25(OH)_2_D_3_ (0.5 μg/kg) and docetaxel significantly increased the period of time-to-progress of pancreatic cancer as compared to treatment by docetaxel alone [75]. Recently, a VDR-alkylating derivative of 1α,25(OH)_2_D_3_, 1α,25-dihydroxyvitamin D_3_-3-bromoacetate (1α,25(OH)_2_D_3_-3-BE), has been studied *in vitro* and was shown to inhibit the growth of several pancreatic cancer cell lines to a greater extent than 1α,25(OH)_2_D_3_ [56,76]. The *in vitro* activity was further accentuated by combining with 5-amino-imidazole-4-carboxamide-1-beta-4-ribofuranoside (AICAR) [76]. Furthermore, a new vitamin D analog, 19-nor-2α-(3-hydroxypropyl)-1α,25(OH)_2_D_3_ (or MART-10) has been shown to be about 1000-fold more active than 1α,25(OH)_2_D_3_ in inhibiting the proliferation of several prostate cancer cell lines *in vitro* [67,77]. Most importantly, MART-10 does not increase serum calcium in animals [78]. Furthermore, MART-10 is more resistant to 24-hydroxylase-mediated degradation pathway and has a lower binding affinity for DBP compared to 1α,25(OH)_2_D_3_, suggesting that this analog would be more bio-available than 1α,25(OH)_2_D_3_ in circulation [67,77]. Given the poor prognosis and little effective therapeutive options against pancreatic cancer, these two new analogs are promising candidates for further pre-clinical studies, and subsequent clinical trials for pancreatic cancer patients.

### 3.5. Epidemiological Studies of Vitamin D and Pancreatic Cancer

Vitamin D status, as determined primarily by solar UVB exposure and dietary intake of vitamin D, has been shown to positively impact on the incidence of prostate, colon and breast cancers in a number of epidemiological studies [22-24]. For pancreatic cancer, two earlier epidemiologic studies published in 2006 reported an inconsistent relationship between pancreatic cancer incidence and vitamin D status [79,80]. However, the death rate of pancreatic cancer has been found to be inversely associated with sun exposure [81-84]. Two recent pooled nested case-control studies conducted by Stoleznberg-Solomon *et al.* failed to confirm the inversed association between circulating concentration of 25(OH)D and risk of pancreatic cancer [85]. However, the same group did confirm a positive association among subjects with low estimated annual residential solar UVB exposure and pancreatic cancer risk [85]. The lack of association between serum 25(OH)D levels and pancreatic cancer risk could be due to that the study utilized a single serum sample obtained years prior to diagnosis for 25(OH)D measurement. It may well be that serum 25(OH)D changed over the years after the measurement. For example, Yin *et al.* conducted case-control studies with zero lag time between diagnosis and serum 25(OH)D measurement, not nested studies, and found an inverse correlation between serum 25(OH)D level and breast cancer [86].Furthermore, Stoleznberg-Solomon *et al.* showed that a high 25(OH)D level exceeding 100 nmol/L (40 ng/mL) would have a two-fold increase in pancreatic cancer incidence (odds ratio = 2.12, 95% confidence interval: 1.23, 3.64) [87]. On the contrary, a report by Mohr SB *et al.* demonstrated an inverse association between UVB irradiation and incidence rates of pancreatic cancer worldwide. They also found that incidence rates were half as high in countries with estimated serum 25(OH)D > 30 ng/ml than in those with ≤ 30 ng/mL [88]. Some other studies also showed inverse relationships between UVB and pancreatic cancer [89-91]. Results from investigating the association of insulin and glucose levels and the development of pancreatic cancer have also indicated a positive association between high insulin and glucose levels and pancreatic cancer [92-95]. Since vitamin D is capable of regulating the synthesis, binding and actions of insulin [96-98], the finding may imply that an inverse relationship between pancreatic cancer incidence and vitamin D status could exist. Due to these contradictory findings, more careful studies with consideration of the impacts of different genotypes of VDR, DBP, and CYP enzymes (vitamin D hydroxylases) and other possible genomic variance, and environmental factors on vitamin D metabolism and functions are necessary to resolve the question whether vitamin D status has a preventive benefit against the development of pancreatic cancer.

## 4. Conclusions

Pancreatic cancer is a devastating disease with a poor five-year survival of 1–4%. Its characteristics of early spread and distant metastasis make it often diagnosed as a late stage disease and unfit for surgical treatment. Traditional chemotherapy and radiotherapy fail to show a significant benefit to the survival of PCA patients. Facing such a dilemma of dealing with advanced PCA for clinicians, developing new regimens against PCA deserves more attention. Vitamin D was originally discovered a century ago as a “vitamin” to treat rickets, and was believed to play a role only in calcium and phosphate homeostasis and bone mineralization. In the mid and late 1960s, it was realized that vitamin D_3_ itself was not active, and required two sequential hydroxylation steps to be activated, first in the liver to produce 25(OH)D_3_, the circulating form, and then in the kidneys to generate 1α,25(OH)_2_D_3_, the active form. In the late 1970s, it was found that VDR was present in many tissues and cell types not involved in calcium and bone metabolism. Later, it was demonstrated that 1α,25(OH)_2_D_3_ had anti-proliferative activity in a variety of normal and cancer cells, and anti-tumor effects in several animal models. These preclinical findings led to clinical trials using 1α,25(OH)_2_D_3_ on cancer patients. However, it was quickly found out that patients developed hypercalcemia and hypercalciuria. Consequently, several thousands of vitamin D analogs were synthesized with an attempt to eliminate/minimize these side effects, and at the same time, to enhance their anti-proliferative activity. In this respect, several analogs have been shown to be promising *in vitro* and in animal experiments. Unfortunately, they failed to offer promising results in early clinical trials with pancreatic cancer patients. Recently, two highly potent analogs of 1α,25(OH)_2_D_3_, namely 1α,25(OH)_2_D_3_-3-BE and MART-10, may offer promising hope to prolong the period of time-to-progression of patients with advanced pancreatic cancer, especially if the treatment is combined with docetaxel.

## References

[1] Jemal A., Siege R., Ward E., Murray T., Xu J., Thun M.J. (2007). Cancer statistics, 2007. CA Cancer J. Clin..

[2] American Cancer Society (2008). Cancer Facts and Figures 2008.

[3] Trede M., Schwall G., Saeger H.D. (1990). Survival after pancreatoduodenectmy. 118 Consecutive resections without an operative mortality. Ann. Surg..

[4] Yeo C.J., Cameron J.L., Sohn T.A., Lillemoe K.D., Pitt H.A., Talamini M.A. (1997). Six hundred fifty consecutive pancreaticoduodenectomies in the 1990s; pathology, complications, and outcomes. Ann. Surg..

[5] Nitecki S.S., Sarr M.G., Colby T.V., Heerden J.A. (1995). Long-term survival after resection for ductal adenocarcinoma of the pancreas. Is it really improving?. Ann. Surg..

[6] Haller D.G. (2003). New perspectives in the management of pancreas cancer. Semin. Oncol..

[7] Raimondi S., Maisonneuve P., Lowenfels A.B. (2009). Epidemiology of pancreatic cancer: An overview. Nat. Rev. Gastroenterol. Hepatol..

[8] Hassan M.M., Bondy M.L., Wolff R.A., Abbruzzese J.L., Vauthey J.N., Pisters P.W., Evans D.B., Khan R., Chou T.H., Lenzi R., Jiao L., Li D. (2007). Risk factors for pancreatic cancer: Case-control study. Am. J. Gastroenterol..

[9] Iodice S., Gandini S., Maisonneuve P., Lowenfels A.B. (2008). Tobacco and the risk of pancreatic cancer: A review and meta-analysis. Langenbecks Arch. Surg..

[10] Rohrmann S., Linseisen J., Vrieling A., Boffetta P., Stolzenberg-Solomon R.Z., Lowenfels A.B., Jensen M.K., Overvad K., Olsen A., Tjonneland A. (2009). Ethanol intake and the risk of pancreatic cancer in the European Prospective Investigation into Cancer and Nutrition (EPIC). Cancer Causes Contr..

[11] Jiao L., Silverman D.T., Schairer C., Thiébaut A.C., Hollenbeck A.R., Leitzmann M.F., Schatzkin A., Stolzenberg-Solomon R.Z. (2009). Alcohol use and risk of pancreatic cancer: The NIH-AARP Diet and Health Study. Am. J. Epidemiol..

[12] Genkinger J.M., Spiegelman D., Anderson K.E., Bergkvist L., Bernstein L., van den Brandt P.A., English D.R., Freudenheim J.L., Fuchs C.S., Giles G.G. (2009). Alcohol intake and pancreatic cancer risk: A pooled analysis of fourteen cohort studies. Cancer Epid. Biomark. Prev..

[13] Reeves G.K., Pirie K., Beral V., Green J., Spencer E., Bull D. (2007). Million Women Study Collaboration. Cancer incidence and mortality in relation to body mass index in the Million Women Study: Cohort study. BMJ.

[14] Fryzek J.P., Schenk M., Kinnard M., Greenson J.K., Garabrant D.H. (2005). The association of body mass index and pancreatic cancer in residents of southeastern Michigan, 1996-1999. Am. J. Epidemiol..

[15] Patel A.V., Rodriguez C., Bernstein L., Chao A., Thun M.J., Calle E.E. (2005). Obesity, recreational physical activity, and risk of pancreatic cancer in a large U.S. Cohort. Cancer. Epidemiol. Biomarkers Prev..

[16] Berrington de Gonzalez A., Sweetland S., Spencer E. (2003). A meta-analysis of obesity and the risk of pancreatic cancer. Br. J. Cancer..

[17] Vrieling A., Verhage B.A., van Duijnhoven F.J., Jenab M., Overvad K., Tjønneland A., Olsen A., Clavel-Chapelon F., Boutron-Ruault M.C., Kaaks R. (2009). Fruit and vegetable consumption and pancreatic cancer risk in the European Prospective Investigation into Cancer and Nutrition. Int. J. Cancer.

[18] Batty G.D., Kivimaki M., Morrison D., Huxley R., Smith G.D., Clarke R., Marmot M.G., Shipley MJ. (2009). Risk factors for pancreatic cancer mortality: Extended follow-up of the original Whitehall Study. Cancer Epidemiol. Biomark. Prev..

[19] Landi S. (2009). Genetic predisposition and environmental risk factors to pancreatic cancer: A review of the literature. Mutat. Res..

[20] Lowenfels A.B., Maisonneuve P. (2006). Epidemiology and risk factors for pancreatic cancer. Best Pract. Res. Clin. Gastroenterol..

[21] Curado M.P., Edwards B.K., Shin H.R., Storm H. (2007). Cancer Incidence in Five Continents.

[22] Garland C.F., Garland F.C. (1980). Do sunlight and vitamin D reduce the likelihood of colon cancer?. Int. J. Epidemiol..

[23] Schwartz G.G., Hulka B.S. (1990). Is vitamin D deficiency a risk factor for prostate cancer? (Hypothesis). Anticancer Res..

[24] Gorham E.D., Garland F.C., Garland C.F. (1990). Sunlight and breast cancer incidence in the USSR. Int. J. Epidemiol..

[25] Vogelstein B., Kinzler K.W. (2004). Cancer genes and the pathways they control. Nat. Med..

[26] Hruban R.H., Maitra A., Goggins M. (2008). Update on pancreatic intraepithelial neoplasia. Int. J. Clin. Exp. Pathol..

[27] Takaori K. (2007). Current understanding of precursors to pancreatic cancer. J. Hepatobiliary. Pancreat. Surg..

[28] Hruban R.H., Adsay N.V., Albores-Saavedra J., Compton C., Garrett E.S., Goodman S.N., Kern S.E., Klimstra D.S., Klöppel G., Longnecker D.S., Lüttges J., Offerhaus G.J. (2001). Pancreatic intraepithelial neoplasia: A new nomenclature and classification system for pancreatic duct lesions. Am. J. Surg. Pathol..

[29] Hruban R.H., Takaori K., Klimstra D.S., Adsay N.V., Albores-Saavedra J., Biankin A.V., Biankin S.A., Compton C., Fukushima N., Furukawa T. (2004). An illustrated consensus on the classification of pancreatic intraepithelial neoplasia and intraductal papillary mucinous neoplasms. Am. J. Surg. Pathol..

[30] Almoguera C., Shibata D., Forrester K., Martin J., Arnheim N., Perucho M. (1988). Most human carcinomas of the exocrine pancreas contain mutant c-K-ras genes. Cell.

[31] Caldas C., Hahn S.A., da Costa L.T., Redston M.S., Schutte M., Seymour A.B., Weinstein C.L., Hruban R.H., Yeo C.J., Kern S.E. (1994). Frequent somatic mutations and homozygous deletions of the p16 (MTS1) gene in pancreatic adenocarcinoma. Nat. Genet..

[32] Redston M.S., Caldas C., Seymour A.B., Hruban R.H., da Costa L., Yeo C.J., Kern S.E. (1994). p53 mutations in pancreatic carcinoma and evidence of common involvement of homocopolymer tracts in DNA microdeletions. Cancer Res..

[33] Hahn S.A., Schutte M., Hoque A.T., Moskaluk C.A., da Costa L.T., Rozenblum E., Weinstein C.L., Fischer A., Yeo C.J., Hruban R.H., Kern S.E. (1996). DPC4, a candidate tumor suppressor gene at human chromosome 18q21.1. Science.

[34] Li C., Heidt D.G., Dalerba P., Burant C.F., Zhang L., Adsay V., Wicha M., Clarke M.F. (2007). Simeone, DM. Identification of pancreatic cancer stem cells. Cancer Res..

[35] Hermann P.C., Huber S.L., Herrler T., Aicher A., Ellwart J.W., Guba M., Bruns C.J., Heeschen C. (2007). Distinct populations of cancer stem cells determine tumor growth and metastatic activity in human pancreatic cancer. Cell Stem Cell..

[36] Neoptolemos J.P., Stocken D.D., Friess H., Bassi C., Dunn J.A., Hickey H., Beger H., Fernandez-Cruz L., Dervenis C., Lacaine F., Falconi M., Pederzoli P., Pap A., Spooner D., Kerr D.J., Büchler M.W. (2004). European Study Group for Pancreatic Cancer. A randomized trial of chemoradiotherapy and chemotherapy after resection of pancreatic cancer. N. Engl. J. Med..

[37] Oettle H., Post S., Neuhaus P., Gellert K., Langrehr J., Ridwelski K., Schramm H., Fahlke J., Zuelke C., Burkart C., Gutberlet K., Kettner E., Schmalenberg H., Weigang-Koehler K., Bechstein WO., Niedergethmann M., Schmidt-Wolf I., Roll L., Doerken B., Riess H. (2007). Adjuvant chemotherapy with gemcitabine vs observation in patients undergoing curative-intent resection of pancreatic cancer: A randomized controlled trial. JAMA.

[38] Regine W.F., Winter K.A., Abrams R.A., Safran H., Hoffman J.P., Konski A., Benson A.B., Macdonald J.S., Kudrimoti M.R., Fromm M.L., Haddock M.G., Schaefer P., Willett C.G., Rich T.A. (2008). Fluorouracil vs gemcitabine chemotherapy before and after fluorouracil-based chemoradiation following resection of pancreatic adenocarcinoma: A randomized controlled trial. JAMA.

[39] Herman J.M., Swartz M.J., Hsu C.C., Winter J., Pawlik T.M., Sugar E., Robinson R., Laheru D.A., Jaffee E., Hruban R.H., Campbell K.A., Wolfgang C.L., Asrari F., Donehower R., Hidalgo M., Diaz L.A., Yeo C., Cameron JL., Schulick RD., Abrams R. (2008). Analysis of fluorouracil-based adjuvant chemotherapy and radiation after pancreaticoduodenectomy for ductal adenocarcinoma of the pancreas: Results of a large, prospectively collected database at the Johns Hopkins Hospital. J. Clin. Oncol..

[40] Renouf D., Moore M. (2010). Evolution of systemic therapy for advanced pancreatic cancer. Expert. Rev. Anticancer Ther..

[41] Mozolowski W., Sniadecki J. (1939). On the cure of rickets. Nature.

[42] Palm T. (1890). The geographical distribution and etiology of rickets. Practitiner.

[43] McCollum E.V., Simmons N., Pitz W. (1916). The relaitn of the unidentified dietary factors, the fat-soluble A and water soluble B of the diet to the growth promoting properties of milk. J. Biol. Chem..

[44] McCollum E.V., Simmond s N., Becker J.E., Shipley P.G. (1922). An experimental demonstratin of the existence of a vitamin which promotes calcium deposition. J. Biol. Chem..

[45] Huldshinsky K. (1919). Heilung von rachitis durch kunstlich hohensonne. Deut. Med. Wochenschr..

[46] Steenbock H. (1924). Black fat-soluble vitamins. XVII. The induction of growth-promoting and calcifying properties in a ration by exposure to ultraviolet light. J. Biol. Chem..

[47] Holick M.F. (2004). Sunlight and vitamin D for bone health and prevention of autoimmune diseases, cancers, and cardiovascular disease. Am. J. Clin. Nutr..

[48] Holick M.F., MacLaughlin J.A., Clark M.B., Holick S.A., Potts J.T., Anderson R.R., Blank I.H., Parrish J.A., Elias P. (1980). Photosynthesis of previtamin D_3_ in human skin and the physiologic consequences. Science.

[49] MacLaughlin J.A., Anderson R.R., Holick M.F. (1982). Spectral character of sunlight modulates photosynthesis of previtamin D_3_ and its photoisomers in human skin. Science.

[50] Segart S. (2008). Vitamin D regulation of cathelicidin in the skin. Toward a renaissance of vitamin D in dermatology?. J. Invest. Dermatol..

[51] Lehmann B., Meurer M. (2010). Vitamin D metabolism. Dermatol. Ther..

[52] Schuster I. (2011). Cytochromes P450 are essential players in the vitamin D signaling system. Biochim Biophys. Acta.

[53] Zehnder D., Bland R., Williams M.C., McNinch R.W., Howie A.J., Stewart P.M., Hewison M. (2001). Extrarenal expression of 25-hydroxyvitamin D_3_-1 alpha-hydroxylase. J. Clin. Endocrinol. Metab..

[54] Omdahl J.L., Morris H.A., May B.K. (2002). Hydroxylase enzymes of the vitamin D pathway: Expression, function, and regulation. Annu. Rev. Nutr..

[55] Chiang K.C., Chen T.C. (2009). Vitamin D for the prevention and treatment of pancreatic cancer. World J. Gastroenterol..

[56] Schwartz G.G., Eads D., Rao A., Cramer S.D., Willingham M.C., Chen T.C., Jamieson D.P., Wang L., Burnstein K.L., Holick M.F., Koumenis C. (2004). Pancreatic cancer cells express 25-Hydroxyvitamin D-1 {alpha}-hydroxylase and their proliferation is inhibited by the prohormone 25-hydroxyvitamin D_3_. Carcinogenesis.

[57] Haussler M.R., Haussler C.A., Jurutka P.W., Thompson P.D., Hsieh J.C., Remus L.S., Selznick S.H., Whitfield G.K. (1997). The vitamin D hormone and its nuclear receptor: Molecular actions and disease states. J. Endocrinol..

[58] Ramagopalan S.V., Heger A., Berlanga A.J., Maugeri N.J., Lincoln M.R., Burrell A., Handunnetthi L., Handel A.E., Disanto G., Orton S.M., Watson C.T., Morahan J.M., Giovannoni G., Ponting C.P., Ebers G.C., Knight J.C. (2010). A ChIP-seq defined genome-wide map of vitamin D receptor binding: Associations with disease and evolution. Genome Res..

[59] Stumpf W.E., Sar M., Reid F.A., Tanaka Y., DeLuca H.F. (1979). Target cells for 1,25-dihydroxyvitamin D_3_ in intestinal tract, stomach, kidney, skin, pituitary, and parathyroid. Science.

[60] Feldman D., Malloy P.J., Krishnan A.V., Balint E., Marcus R., Feldman D., Nelson D.A., Rosen C.J. (2008). Vitamin D: Biology, action, and clinical implications. Osteoporosis.

[61] Adams J.S., Hewison M. (2010). Update in vitamin D. J. Clin. Endocrinol. Metab..

[62] Bikle D. (2009). Nonclassic actions of vitamin D. J. Clin. Endocrinol. Metab..

[63] Colston K., Hirt M., Feldman D. (1980). Organ distribution of the cytoplasmic 1,25-dihydroxycholecalciferol receptor in various mouse tissues. Endocrinology..

[64] Chen T.C., Holick M.F. (2003). Vitamin D and prostate cancer prevention and treatment. Trends Endocrinol. Metab..

[65] Skowronski R.J., Peehl D.M., Feldman D. (1993). Vitamin D and prostate cancer: 1,25-Dihydroxyvitamin D_3_ receptors and actions in human prostate cancer cell lines. Endocrinology.

[66] Chiang K.C., Persons K.S., Istfan N.W., Holick M.F., Chen T.C. (2009). Fish oil enhances the antiproliferative effect of 1alpha,25-dihydroxyvitamin D_3_ on liver cancer cells. Anticancer Res..

[67] Flanagan J.N., Zheng S., Chiang K.C., Kittaka A., Sakaki T., Nakabayashi S., Zhao X., Spanjaard R.A., Persons K.S., Mathieu J.S., Holick M.F., Chen T.C. (2009). Evaluation of 19-nor-2alpha-(3-hydroxypropyl)-1alpha,25-dihydroxyvitamin D_3_ as a therapeutic agent for androgen-dependent prostate cancer. Anticancer Res..

[68] Kawa S., Nikaido T., Aoki Y., Zhai Y., Kumagai T., Furihata K., Fujii S., Kiyosawa K. (1997). Vitamin D analogues up-regulate p21 and p27 during growth inhibition of pancreatic cancer cell lines. Br. J. Cancer.

[69] Schwartz G.G., Eads D., Naczki C., Northrup S., Chen T.C., Koumenis C. (2008). 19-nor-1 alpha,25-dihydroxyvitamin D_2_ (paricalcitol) inhibits the proliferation of human pancreatic cancer cells *in vitro* and *in vivo*. Cancer Biol. Ther..

[70] Kawa S., Yoshizawa K., Tokoo M., Imai H., Oguchi H., Kiyosawa K., Homma T., Nikaido, Furihata K. (1996). Inhibitory effect of 220-oxa-1,25-dihydroxyvitamin D_3_ on the proliferation of pancreatic cancer cell lines. Gastroenterology.

[71] Colston K.W., James S.Y., Ofori-Kuragu E.A., Binderup L., Grant A.G. (1997). Vitamin D receptors and anti-proliferative effects of vitamin D derivatives in human pancreatic carcinoma cells *in vivo* and *in vitro*. Br. J. Cancer.

[72] Pettersson F., Colston K.W., Dalgleish A.G. (2000). Differential and antagonistic effects of 9-cis-retinoic acid and vitamin D analogues on pancreatic cancer cells *in vitro*. Br. J. Cancer.

[73] Zugmaier G., Jäger R., Grage B., Gottardis M.M., Havemann K., Knabbe C. (1996). Growth-inhibitory effects of vitamin D analogues and retinoids on human pancreatic cancer cells. Br. J. Cancer.

[74] Evans T.R., Colston K.W., Lofts F.J., Cunningham D., Anthoney D.A., Gogas H., de Bono J.S., Hamberg K.J., Skov T., Mansi J.L. (2002). A phase II trial of the vitamin D analogue Seocalcitol (EB1089) in patients with inoperable pancreatic cancer. Br. J. Cancer.

[75] Blanke C.D., Beer T.M., Todd K., Mori M., Stone M., Lopez C. (2009). Phase II study of calcitriol-enhanced docetaxel in patients with previouslyuntreated metastatic or locally advanced pancreatic cancer. Invest. New Drugs.

[76] Persons K.S., Eddy V.J., Chadid S., Deoliveira R., Saha A.K., Ray R. (2010). Anti-growth effect of 1,25-dihydroxyvitamin D_3_-3-bromoacetate alone or in combination with 5-amino-imidazole-4-carboxamide-1-beta-4-ribofuranoside in pancreatic cancer cells. Anticancer Res..

[77] Chen T.C., Persons K.S., Zheng S., Mathieu J., Holick M.F., Lee Y.F., Bao B., Arai M.A., Kittaka A. (2007). Evaluation of C-2-substituted 19-nor-1α,25-dihydroxyvitamin D_3_ analogs as therapeutic agents for prostate cancer. J. Steroid Biochem. Mol. Biol..

[78] Iglesias-Gato D., Zheng S., Flanagan J.N., Jiang L., Sakaki T., Nakabayashi S., Kittaka A., LeBrasseur N.K., Norstedt G., Chen T.C. (2010). The C-2 substituted 19-nor-1α,25-dihydroxyvitamin D_3_ analog, MART-10, has enhanced chemotherapeutic potency in PC-3 prostate cancer cells..

[79] Skinner H.G., Michaud D.S., Giovannucci E., Willett W.C., Colditz G.A., Fuchs C.S. (2006). Vitamin D intake and the risk for pancreatic cancer in two cohort studies. Cancer Epidemiol. Biomarkers Prev..

[80] Stolzenberg-Solomon R.Z., Vieth R., Azad A., Pietinen P., Taylor P.R., Virtamo J., Albanes D. (2006). A prospective nested case-control study of vitamin D status and pancreatic cancer risk in male smokers. Cancer Res..

[81] Grant W.B. (2007). An ecologic study of cancer mortality rates in Spain with respect to indices of solar UVB irradiance and smoking. Int. J. Cancer.

[82] Boscoe F.P., Schymura M.J. (2006). Solar ultraviolet-B exposure and cancer incidence and mortality in the United States, 1993-2002. BMC Cancer.

[83] Tuohimaa P., Pukkala E., Scélo G., Olsen J.H., Brewster D.H., Hemminki K., Tracey E., Weiderpass E., Kliewer E.V., Pompe-Kirn V., McBride M.L., Martos C., Chia K.S., Tonita J.M., Jonasson J.G., Boffetta P., Brennan P. (2007). Does solar exposure, as indicated by the non-melanoma skin cancers, protect from solid cancers: Vitamin D as a possible explanation. Eur. J. Cancer.

[84] Mizoue T. (2004). Ecological study of solar radiation and cancer mortality in Japan. Health Phys..

[85] Stolzenberg-Solomon R.Z., Hayes R.B., Horst R.L., Anderson K.E., Hollis B.W., Silverman D.T. (2009). Serum vitamin D and risk of pancreatic cancer in the prostate, lung, colorectal, and ovarian screening trial. Cancer Res..

[86] Yin L., Grandi N., Raum E., Huang U., Arndt V., Brenner H. (2010). Meta-analysis: Serum vitamin D and breast cancer risk. Eur. J. Cancer.

[87] Stolzenberg-Solomon R.Z., Jacobs E.J., Arslan A.A., Qi D., Patel A.V., Helzlsouer K.J., Weinstein S.J., McCullough M.L., Purdue M.P., Shu X.O., Snyder K. (2010). Circulating 25-hydroxyvitamin D and risk of pancreatic cancer: Cohort Consortium Vitamin D Pooling Project of Rarer Cancers. Am. J. Epidemiol..

[88] Mohr S.B., Garland C.F., Gorham E.D., Grant W.B., Garland F.C. (2010). Ultraviolet B irradiance and vitamin D status are inversely associated with incidence rates of pancreatic cancer worldwide. Pancreas.

[89] Giovannucci E., Liu Y., Rimm E.B., Hollis B.W., Fuchs C.S., Stampfer M.J., Willett W.H. (2006). Prospective study of predictors of vitamin D status and cancer incidence and mortality in men. JNCI.

[90] Kato I., Tajima K., Kuroishi T., Tominaga S. (1985). Latitude and pancreatic cancer. Jpn. J. Clin. Oncol..

[91] Neale R.E., Youlden D.R., Krnjacki L., Kimlin M.G., van der Pols J.C. (2009). Latitude variation in pancreatic cancer mortality in Australia. Pancreas.

[92] Stolzenberg-Solomon R.Z., Graubard B.I., Chari S., Limburg P., Taylor P.R., Virtamo J., Albanes D. (2005). Insulin, glucose, insulin resistance, and pancreatic cancer in male smokers. JAMA.

[93] Huxley R., Ansary-Moghaddam A., Berrington de González A., Barzi F., Woodward M. (2005). Type-II diabetes and pancreatic cancer: A meta-analysis of 36 studies. Br. J. Cancer.

[94] Michaud D.S., Wolpin B., Giovannucci E., Liu S., Cochrane B., Manson J.E., Pollak M.N., Ma J., Fuchs C.S. (2007). Prediagnostic plasma C-peptide and pancreatic cancer risk in men and women. Cancer Epidemiol. Biomarkers Prev..

[95] Hennig R., Ding X.Z., Adrian T.E. (2004). On the role of the islets of Langerhans in pancreatic cancer. Histol. Histopathol..

[96] Mathieu C., Gysemans C., Giulietti A., Bouillon R. (2005). Vitamin D and diabetes. Diabetologia.

[97] Maestro B., Campión J., Dávila N., Calle C. (2000). Stimulation by 1,25-dihydroxyvitamin D_3_ of insulin receptor expression and insulin responsiveness for glucose transport in U-937 human promonocytic cells. Endocr. J..

[98] Maestro B., Dávila N., Carranza M.C., Calle C. (2003). Identification of a Vitamin D response element in the human insulin receptor gene promoter. J. Steroid Biochem. Mol. Biol..

